# Making the difficult career transition: Writing the next chapter during the great resignation or in the future

**DOI:** 10.3389/fpsyg.2022.905813

**Published:** 2022-10-25

**Authors:** Paul J. Coppola, Aprille F. Young

**Affiliations:** Department of Organizational and Leadership Psychology, William James College, Newton, MA, United States

**Keywords:** adaptive leadership and change, branding, career change, career transition, development, personal branding, great resignation

## Abstract

During the midst of the Great Resignation, over 4.5 million people have changed jobs. While a job change does not register as one of the top three drivers of stress, career transition-related stress does present itself as one of the top 25 causes. This stress can be reduced through social support models, career transition planning, and personal brand strategy frameworks. These adaptive change models become part of a continuous learning and growth process. This literature review aims to contribute to the industry and define career transition through a more holistic personal brand strategy utilizing a wide range of disciplines considering the challenges and opportunities presented during the Great Resignation.

## Introduction – The impetus for career change

During the first wave of COVID-19, in early 2020, the Bureau of Labor Statistics stated that most employees stayed in their jobs due to (quarantine) lockdowns and the uncertainty of impending layoffs (Serenko, 2022). This behavior began to radically shift in 2021 as the economic recovery started, businesses began reopening and increasing their capacity, and new skilled and knowledge jobs appeared at an unprecedented rate. According to The Work Trend Index (2021), 46% of the career transitioning population decided to make changes to jobs outside of their industry or field (Serenko, 2022). Starting with what would be coined the Great Resignation, 4.5 million people per month or nearly 3% of the workforce voluntarily resigned from their job and/or company in the hopes of finding a different role or changing their career path completely (Davidson, 2022).

**Figure 1:**
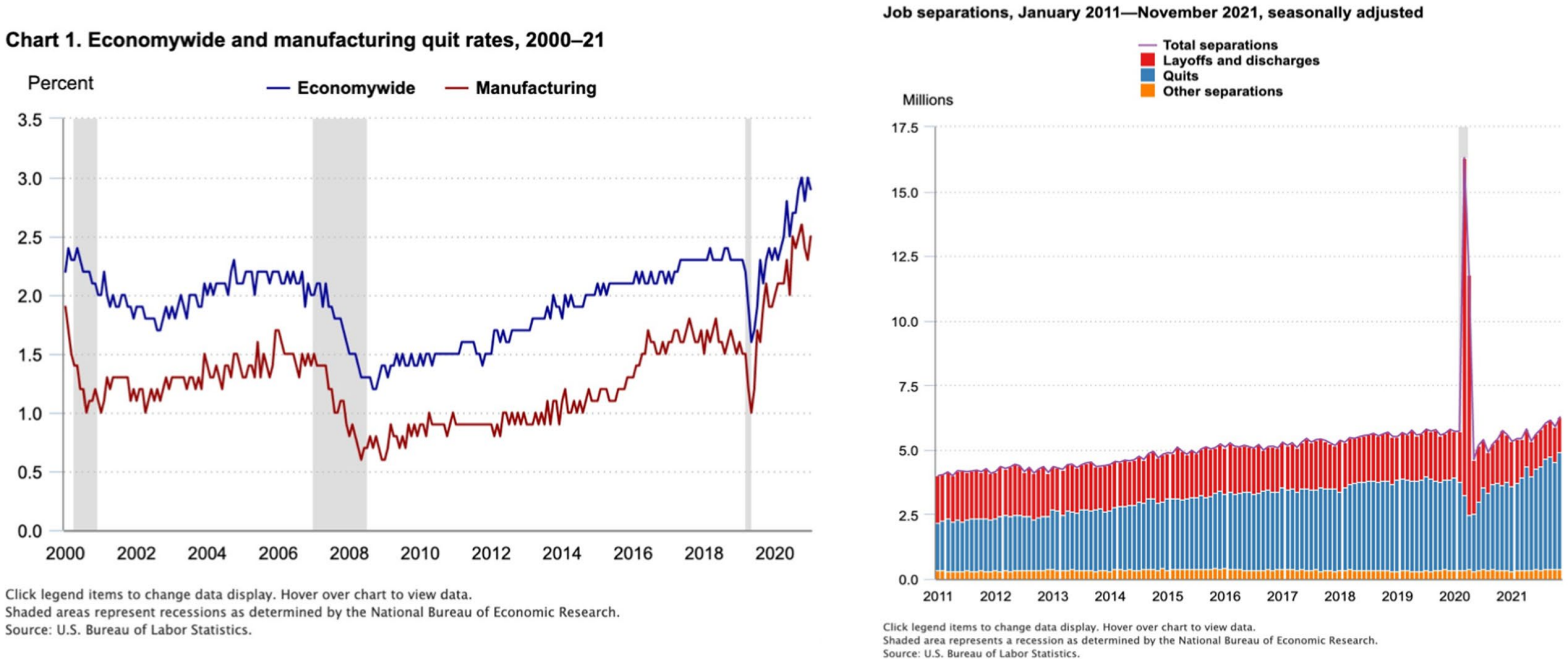
Economywide and manufacturing quit rates. Represents the highest voluntary separation rate (quits) in 2021. In November 2021, quits increased in several industries, with the largest increases in accommodation and food services; health care and social assistance; and transportation, warehousing, and utilities (The Bureau of Labor Statistics, 2022).

Career is defined as a set of events or experiences occurring over a person’s work life (Rensburg and Ukpere, 2014). In 2021, employers began posting nearly 10.6 million jobs in an average month creating a worldwide shortage for talent (Davidson, 2022). The Great Resignation was coined by Dr. Anthony Klotz of Texas A&M (at the time) as the mass exodus of employees leaving their jobs in search of new opportunities (Serenko, 2022). The Great Resignation in its own way is an impetus to an organizing principle which explains a carefully choreographed phenomenon or ‘perfect storm’ that created an extensive labor shortage and introduced significant implications for how organizations serviced their customers, especially those in knowledge intensive, service, healthcare, and manufacturing industries where attrition rates were at an all-time high (see Figure 1) and surged anywhere from 2 to 40% (Serenko, 2022; The Bureau of Labor Statistics, 2022). The Great Resignation also accelerated this transition to a knowledge economy, or a consumption system that is based on intellectual capital (Serenko, 2022). This shift required potential job candidates to rebrand themselves in advance of making their desired career transition (Gorbatov et al., 2018, 2019). Although the Parker and Menasce Horowitz (2022) initially identified low pay as the main driver and dissatisfaction in the workplace environment, the stress of the COVID-19 pandemic created a space for employees to step back and reevaluate their purpose and accomplishments, their work environment, and their career path (Serenko, 2022).

These new opportunities did lead to increased pay (Serenko, 2022), the ability to step out of the workforce altogether whether the individual intended to return at some point or retire, an attempt to ‘reduce’ personal stress (Sullivan and Al Ariss, 2019; Parker and Menasce Horowitz, 2022), create a mechanism to leave behind toxic or dysfunctional work cultures (Sull et al., 2022), and in many instances, pursue and transition into different types of jobs and careers (Business Information Review, 2022; Serenko, 2022). Regardless of the rationale behind the choice to leave or transition, there was hope of finding renewed purpose (George and Sims, 2007) that better aligned with one’s values. This required that job seekers ‘reinvent’ themselves and reevaluate their personal brand (Gorbatov et al., 2018; Sull et al., 2022). This transition resulted in job candidates crossing “the boundaries of occupations, industries, organizations, functional areas, countries, and labor markets” (Sullivan and Al Ariss, 2021, p. 1).

## Making career transitions

Career transition is defined as the period during which an individual shifts to a different work setting or role (Greer and Kirk, 2022). Additionally, Sullivan and Ariss (2018) explain that this period of transition is where an individual is either changing roles, taking on a different objective, or realigning to a different space (Sullivan and Ariss, 2018). Transition traverses across different types of boundaries regardless of how major or minor it can be for the individual (Rensburg and Ukpere, 2014; Masdonati et al., 2022). These boundaries or experiences can include a change of job, company, industry, or even stepping out of the workforce altogether. Rensburg and Ukpere (2014) describe these transitions as either inter-role (i.e., between new and different roles) or intra-role (i.e., which introduces change over time). For the purposes of this literature evaluation, the authors focused on both inter- and intra-role changes that include career transitions to different types of roles and industries.

Masdonati et al. (2022) state that people tend to go through several career transitions throughout their lives due to life changes, loss of job, change of interests, health- and family-related issues, or through the acquisition of new skills (Dawson et al., 2021). This was also personified by the added pressures of the COVID-19 pandemic. This type of change comes with stress, anxiety, uncertainty, regret, self-esteem-related issues, and what can feel like extenuating waiting periods (Veisani et al., 2021). This type of transition, however, can also restoratively be coupled with hope, excitement, renewed energy, and even happiness as it relates to accomplishments and success (Walsh et al., 2018; Öztemel and Yıldız-Akyol, 2021). Boehm and Lyubomirsky posit that there is a direct correlation between happiness and career success (Walsh et al., 2018). They describe such examples as happier people receiving higher ratings and compensation due to stronger performance (Walsh et al., 2018).

Career transition is more than just changing jobs, vertically integrating into the same industry, or even moving to a different organization that performs similar activities. It includes moving to a new industry altogether or shifting career paths (Rensburg and Ukpere, 2014; Sullivan and Ariss, 2018). It can also include redefining through a new brand, skills, or education, becoming self-employed, or taking on a role that, on the surface, may not look like a natural career progression (Gorbatov et al., 2019). In addition, the initial trigger for career transition may not always be welcomed or planned. This change can be a result of a job elimination, forced role change due to reorganization, or a move to avoid becoming obsolete (Masdonati et al., 2022).

While a job change does not rise among the top three primary drivers of stress, job change-related stress does present itself as one of the top 25 causes (Davidson, 2022). Stress is the body’s response to any demands, changes, or threats (Veisani et al., 2021). Although the cause of stress and stressors may be similar across individuals, individuals may perceive or react to the stress differently based on their personality traits, anxiety levels, and levels of self-esteem (Veisani et al., 2021). For example, barriers to rebranding and making changes may feel insurmountable to one individual and can be easily overcome by another depending on their motivation levels and adaption skills (Rensburg and Ukpere, 2014; Veisani et al., 2021). Well-organized personal branding and career transition strategies, however, can act as a coping mechanism and stabilizing factor to reduce what feels like an insurmountable barrier and create more adaptive and growth mindset mechanisms during stressful career transitions (Rensburg and Ukpere, 2014).

Individuals who are more adaptive, or demonstrate a growth mindset (Dweck, 2019), tend to acquire new skills and can make the necessary connections to achieve successful job shifts (Öztemel and Yıldız-Akyol, 2021). Öztemel and Yıldız-Akyol (2021) further describe that happiness, perceived social support, and a positive and planful attitude toward the future, are correlated with career adaptability. Career transition and adaptability, whether voluntary, such as job changes, or involuntary, such as layoffs, presents opportunities to grow through uncertain, challenging periods (Masdonati et al., 2022). Conversely, those individuals who possess more of a fixed mindset (Dweck, 2019) and struggle to see their own ability to adapt and grow in times of transition, may find it difficult to overcome such challenges and reach their career transition goals (Masdonati et al., 2022).

### Strategic career shifts

Baruch (2004) states that a career is a process of development journey based on experiences and jobs held in different organizations (Baruch and Rosenstein, 1992). Career path, field, or inter-role career changes are less typically motivated by salary and tend to be driven by one’s values, purpose, and aspirations, the need for job security, and desire for satisfaction (George and Sims, 2007; Uy, 2020). According to Gibbons et al. (2011), the most successful career switchers strategically plan, taking years to learn new skills, network, and prepare themselves financially to make a move (Uy, 2020). Making this type of career change is often validated by the significant amount of time and effort put in to carry out this important strategic goal. As a result of the complexities, people may postpone or avoid these types of decisions due to timing, lack of commitment, prioritizing life’s demands (e.g., caregiving, education, needs over happiness), or lack of confidence in one’s ability and skills to make the change (Uy, 2020; Öztemel and Yıldız-Akyol, 2021). It is a decision that requires personal reflection back to goals, purpose, and values (George and Sims, 2007). Some individuals sit with the decision for many years contemplating what this transition could look like. Other people postpone until the ‘right time,’ which throughout a lifetime may never come. Most recognize embarking upon this ideal career journey, may require (1) setting a personal strategy and plan (George and Sims, 2007), (2) understanding one’s personal risk tolerance (Baruch, 2004), (3) defining which priorities are most important (Baruch, 2004), (4) having a framework of must-haves, nice-to-haves, and unnecessary components, and (5) narrowing down what can be sacrificed even temporarily. For those individuals who may be more risk adverse or reluctant for any number of personal reasons, such as family, financial commitments, known obstacles, fear of failure, and discomfort with the unknown, the personal career transition and personal branding plan may need to be more conservative.

### Personal branding

Personal Branding is the “strategic process of creating, positioning, and maintaining a positive impression of oneself, based on a unique combination of individual characteristics, which signal a certain promise to the target audience through a differentiated narrative and imagery” (Gorbatov et al., 2019, p. 1). Personal branding allows for “self-branding” in a knowledge economy as a mechanism of self-promotion in pursuit of self-realization (Gandini, 2016; Gorbatov et al., 2019). This process is strategic in that it is coordinated and differentiated at the individual level (Gorbatov et al., 2019). While typically proactive, personal branding is a process of both conscious and unconscious behavior (Bolino et al., 2016). This means that an individual can be intentional in their actions to drive a specific perception or may be unintentionally labeled based on demonstrated biases, such as reactions to specific experiences (Bolino et al., 2016). Gorbatov et al. (2019) demonstrated in their research that personal branding had a positive and indirect, yet significant, effect on career satisfaction and career achievement.

## The personal branding strategy model

As Brené Brown said in her book *Dare to Lead*, in order “to be the person who we long to be, we must again be vulnerable. We must take off the armor, put down the weapons, show up, and let ourselves be seen” (Brown, 2018). Brown (2018) also talks about daring leadership. In this context, being a leader is not just defined by a role but rather one’s behavior and mindset. Daring leadership is made up of four skill sets that are teachable, observable, and measurable: (1) rumbling with vulnerability, (2) living into our values, (3) braving trust, and (4) learning to serve (George and Sims, 2007; Brown, 2018). Each of these factors enables us to be open to what comes next, learn, and prepare us to define what is possible. In essence, the career transition process is one of vulnerability, authenticity, and personal reflection on goals, fears, personal limitations, talents, and even biases (Rensburg and Ukpere, 2014; Uy, 2020). While it is also impacted by personal factors, such as career choice and experiences, personality traits, such as openness to motivation and flexibility, are essential (Uy, 2020). These qualities can propel the process forward to achieving the ultimate career transition goal due to a growth mindset (Dweck, 2019). This aligns closely with Pawar’s three C’s model of (1) demonstrating credibility and integrity, (2) observing consistency and the ability to always deliver value, and (3) ensuring **clarity** in purpose and meaning are consistent (Pawar, 2016).

## Getting started

**Figure 2:**
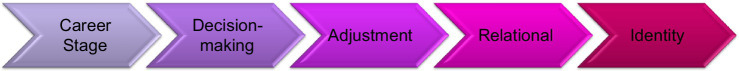
Five major theoretical perspectives of career transitions. While career stage perspectives can be fairly predictable events, transitions may occur as an individual ages and moves from one period of development to another across their career. Research has looked closely at what factors may influence the decision-making process and are affected by feelings of job insecurity, which can be triggered by changing needs within the workforce and flexibility within the role and organization. People transition or adjust toward career transitions over time. Changes in the labor market can further shift thinking and influence the path people may ultimately choose. From the relational perspective, career transitions are socially embedded. Studies have examined how various people in and outside of a persons’ work domain have influenced an individual’s decisions, engagement, and their ability to successfully make career transitions. Identity relies heavily on the individual’s brand and how confident they are in carrying out the transition (Sullivan and Ariss, 2018).

Making a career change starts with five key principles (see Figure 2), (1) look at your career stage and understand your motivation, (2) make and commit to the decision to change, (3) make the necessary adjustments, (4) relate the change to what is important, and (5) establish an identity and a brand purpose (Sullivan and Ariss, 2018).

Individuals who may be more tenured in their career or role may be asked to move away from what they know how to do well and risk moving beyond their ‘frontier of competence’ as they try to respond adaptively to new demands in their environments (Heifetz et al., 2009). For some, there could be a natural resistance toward losing what they are most familiar with. It is key to understand the kinds of personal losses at stake in this changing situation, from the job itself to wealth, status, relationships, relevance, brand identity, community, loyalty, and competence (Heifetz et al., 2009).

### Looking at career stage

**Figure 3:**
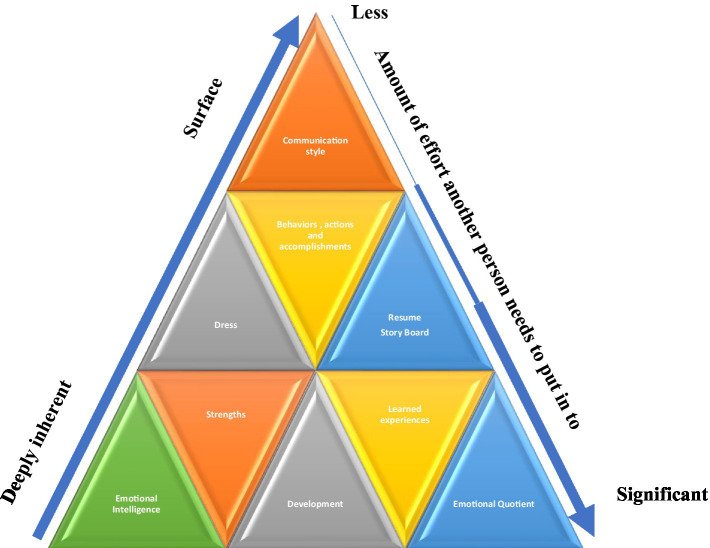
Personal Brand Triangle. The brand triangle represents the make-up of a personal and professional brand and necessary components to ensure it is not confusing to others. Features at the bottom are more deeply rooted within the individual and take longer for others to discover, gradually increasing the amount of required effort to learn more about the person’s brand.

Evaluating the career stage becomes as much of a self-reflective exercise to understand motivations and career aspirations as it does an evaluation of skills and competencies. It also requires setting a career transition strategy framework (see Figure 3) to set end outcomes or goals, education, learning edges, experience requirements, type of support needed (Greer and Kirk, 2022), and realistic timing expectations to execute the plan. It also requires personal reflection on what will make the individual happy (Uy, 2020; Öztemel and Yıldız-Akyol, 2021) and an understanding of what they enjoy doing so much that they would consider doing for ‘free’.

### Make and commit to the decision to change

**Figure 4:**
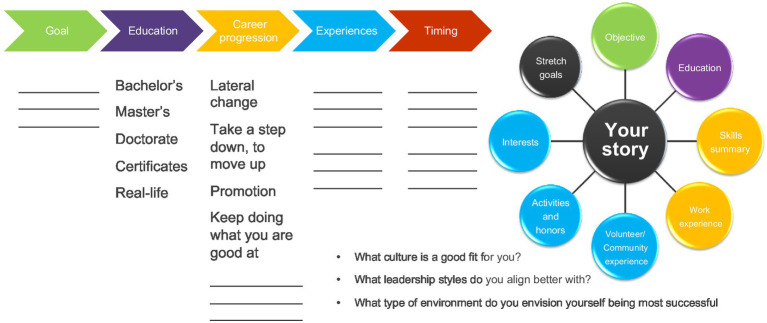
The use of a career transition strategy framework can provide you with a well-thought-through plan that lays out what is most important starting with the goals and desired outcome, working through the plan on how to best achieve. It also helps you to understand the non-negotiables. The non-negotiables become important when stress and anxiety increase, and confidence is in question. It also helps to keep you deeply grounded in your purpose. Personal Brand Triangle.

Starting with a career transition strategy framework (see Figure 4) solidifies the plan of achieving the goals (Gorbatov et al., 2019). In some instances, it may require a combination of solutions to reach the desired outcome. Having this strategy, revisiting it once or twice during the year, and ongoing planning with milestones and measures becomes important for personal accountability and achievement.

### Make the necessary adjustments

Those who have successfully reinvented themselves over their lifetime may feel a career transition is somewhat predictable. However, those who are attempting this change for the first time may require additional support and coaching from colleagues, other contacts in their network, mentors, and even career sponsors to achieve their goals. Transitions occur as an individual ages and moves from one period of development to another across their career. Sullivan and Crocitto’s (2007) research has shown that the career change process can be easier for people who are earlier in their career as organizational practice tends to focus career stage learning and development efforts on this population versus those in later career. Elements like coaching and mentoring create a natural accountability toward goals, timeframes, and approaches allowing the individual to make the adjustments needed (Seehusen et al., 2021). This is an iterative process that requires measurement and re-evaluation.

### Relate the change to what is important

Planning for a career move should be contemplated months or even years before it happens (George and Sims, 2007; Gorbatov et al., 2019). It is important to set a True North or North Star to drive toward while remaining consistent and committed to an endpoint (George and Sims, 2007). The activation of the career strategy transition ramp-up accelerates this work once the pre-planning work has been completed.

## Establish an identity and a brand purpose

Warren Bennis asks the question, “How can you best express you?” He states that there are three tests to examine, (1) knowing what you want, your abilities and capacities, (2) knowing what drives you and gives you satisfaction, and (3) knowing what your values and priorities are (Bennis, 2009).

These three elements help to form a personal brand. Personal brand is made up of both professional and personal elements that define who someone is (see Figure 3), and how they may present themselves consciously and unconsciously (Bolino et al., 2016). For example, there are more visible themes such as communication style (Pawar, 2016; Gorbatov et al., 2019), behavior, appearance (Pawar, 2016), and personal and professional accomplishments (Gorbatov et al., 2019). Less visible external qualities such as emotional intelligence and emotional quotient may require deeper relationships for people to detect (Gorbatov et al., 2019). Emotional Intelligence (EI) is how we handle our emotions (Srivastava, 2013). Emotional quotient (EQ) or also known as emotional intelligence quotient, measures the level of one’s emotional intelligence (Thomas and Inkson, 2009; Srivastava, 2013). EI and EQ particularly become important in self-reflection, self-awareness, and interpersonal engagements with others (Thomas and Inkson, 2009).

Brand positioning requires being clear with others so they can make the necessary connections to accomplishments and qualifications, what capabilities the candidate can offer, and how the incumbent may help the organization to achieve their goals (Pawar, 2016; Gorbatov et al., 2019). Brand also showcases that which inspires an individual through their purpose (George and Sims, 2007) and the impact they have had (Pawar, 2016). What people do not always realize is that brand influences how others are perceived, whether it is intentional or not (Thomas and Inkson, 2009).

## Preparing to make the change

As Carl Gustav Jung had said, “I am not what happened to me, I am what I choose to become.” Professional and personal experiences build upon what an individual has accomplished and should be able to be mapped to the required capabilities needed down the road. Mapping experience and skills to known gaps focus on existing strengths versus educational and experience development opportunities. Learning is a continuous process that progresses over a lifetime. It does not need to be done all at once, and through a carefully appointed career transition plan can be planned out with achievable milestones (Rensburg and Ukpere, 2014). Carrying out the change requires a series of steps, including: (1) establishing a networking plan, (2) defining purpose, and (3) level-setting expectations of persistence, patience, positivity, and professionalism.

### Creating a networking plan

Starting off with a networking stakeholder map determines the most impactful contacts one should connect with during their journey. The network can be activated based on the alignment of set goals and career desires, ensuring conversations can be aligned and maximized with the right people at the right time.

### Relationships

**Figure 5:**
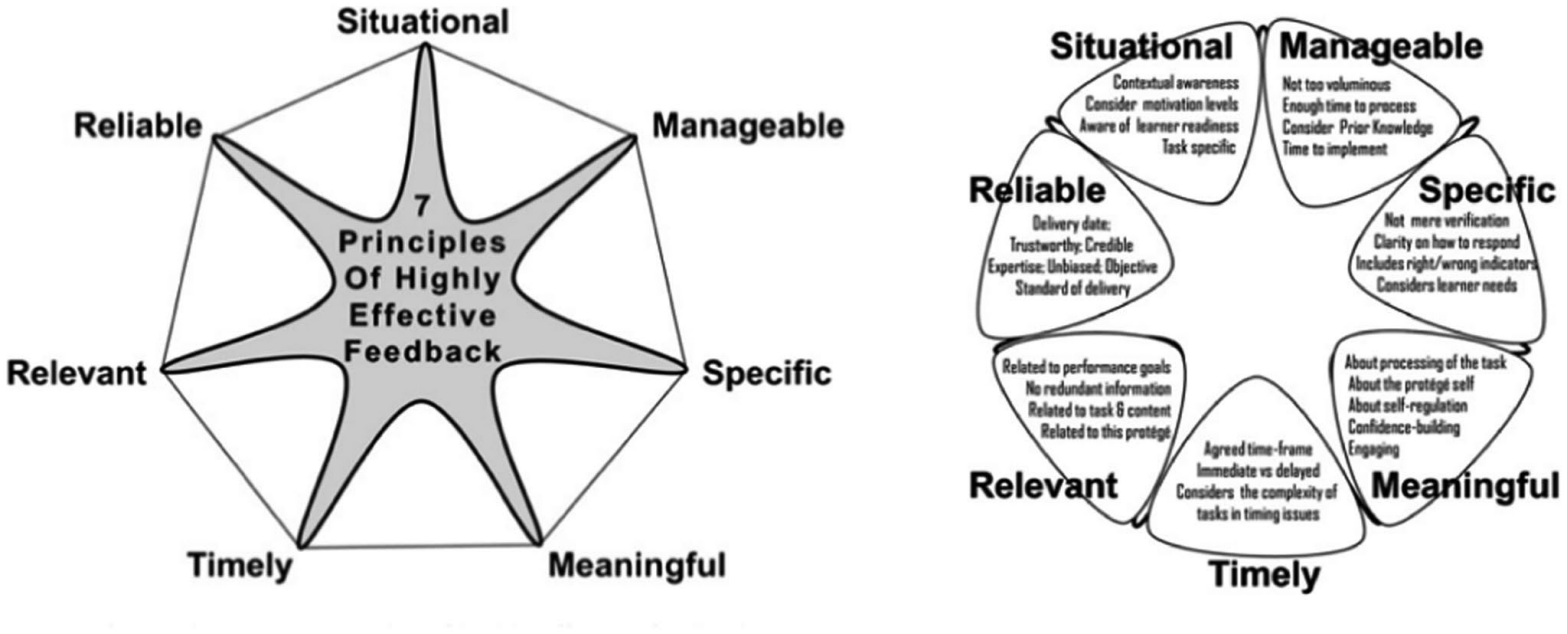
7 Principles of Highly Effective Feedback and Star Performance. Describes the seven principles of highly effective feedback. When adapted to career transitions, it becomes a meaningful way to provide the career switcher with objective and trusted guidance (De Villiers, 2013).

Individuals who successfully make career transitions lean into their current and previous relationships while planning for other future networking connections to close knowledge gaps. These connections elevate prior experiences, enhance social support, and position for a mentor, coaching, and sponsorship relationships (Masdonati et al., 2022). These relationships, when structured with an objective and trusted colleague, can provide valuable feedback on perceptions and future direction to take. De Villiers (2013) describe the power that feedback can offer through the lens of seven principles (see Figure 5). Her posit that feedback should be (1) relevant to the person and the transition, (2) manageable (achievable), (3) specific, (4) meaningful in how it can aid development, (5) timely, (6) from a reliable and trustworthy source, and (7) applicable and connected to the situation from the trusted coach so it can instill confidence (Hattie and Timperley, 2007; De Villiers, 2013). When this model is adapted to career transitions and complemented with personal reflection, it becomes a meaningful way to provide the individual with objective guidance (De Villiers, 2013).

### Mentoring, coaching, and sponsorship

A mentor is an individual who can act as a role model, someone to share ideas with, point out blind spots, collaborate on ideas, and can support development and growth (Seehusen et al., 2021). A coach enables individuals to recognize their capability and acts more deliberately by providing performance and behavioral guidance as a periodic practice (Seehusen et al., 2021). A sponsor, however, acts as a career advocate. They act in a capacity that voices the value the person brings and navigates them toward the career transition goal (Seehusen et al., 2021). One individual can act in the role of all three, or different individuals may play these roles in a career transition.

### Defining purpose

**Figure 6:**
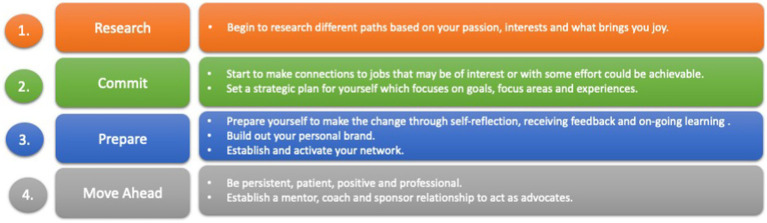
Making the Difficult Transition Checklist. While making a career transition can be challenging, following these steps can help to make the process smoother.

A true north is an inner sense, or a calling, of what someone wants to accomplish in life. It is a combination of values, beliefs, and purpose. It keeps people on a track that is true to them (George and Sims, 2007). George and Sims (2007) argues that it is different for every person. Prior to conducting purpose-driven discussions, individuals can design a conversation guide (see Figure 6) that focuses on a few key areas of interest related to their goals, career progression, and development opportunities. As a result, through a clearly articulated goal and brand statement, people can create a halo effect of how they want to be perceived.

### Self-reflection and authenticity

George and Sims (2007) describes the importance of knowing one’s authentic self. The authenticity process starts with self-reflection and leads to a state of knowing oneself (George and Sims, 2007). George and Sims (2007) suggests that as an individual knows oneself, they will find the passion that motivates them and defines their purpose. The self-reflection process looks deeper at motivations, biases, and behavioral triggers. Self-reflection requires deep introspection and a keen awareness of where energy and fears are generated from. Negativity, for example, can be misread by others as a lack of motivation and empathy (Boyatzis, 2014).

### P-squared: Persistence, patience, positivity, and professionalism

Persistence does not imply irrationality but does require consistency, commitment, and patience in the career transition process. While the process of making a career transition can be fatiguing, as popular articles such as HBR and Forbes claim, it can take anywhere from 5 months to 5 years to make a successful transition depending on the individual and their motivation. By staying positive, positive psychology data has shown that individuals are more successful in obtaining positions and adapting to career transitions (Boyatzis, 2014; Buyukgoze-Kavas, 2016). Studies have also indicated that there are moderate to strong correlations between participants who are more resilient, hopeful, and optimistic as they are more likely to perceive themselves as more agile in their careers (Buyukgoze-Kavas, 2016). According to Boyatzis (2014), negative or dissonant behaviors can cause people to want to avoid someone versus positive or resonant behaviors creating an energized response. While staying positive can be difficult, through self-care and resilience, practicing mindfulness, and reflecting on all one has to offer, the positive psychology framing can also help to stimulate increased professionalism which ensures remaining true to personal brand (George and Sims, 2007).

## Assessment for transition readiness

During this research, the authors identified a 40-item multidimensional Career Transitions Inventory (CTI) which looks at five factors including (a) readiness, (b) confidence, (c) perceived support, (d) control, and (e) decision independence (Heppner et al., 1994). This tool provides an additional layer to examine one’s readiness and potential barriers for this level of career change in the coaching process.

## Limitations and future research

While this literature evaluation examined career transition and personal branding approaches in a changing and constantly evolving job market such as what has been witnessed in the Great Resignation, there is further opportunity to explore additional approaches that can be applied when there is an increase in the jobless rate and decrease in talent demand. This market shift does not imply any less importance on personal brand and career transition strategy and, in fact, may require an additional layer of change planning. Based on the review of the literature, there is also an opportunity to focus more heavily on individuals making transitions into full retirement or moving into self-employment and the value of a personal brand and career transition strategy. Also, at the time of this article, one leadership style over another did not increase the likelihood of successfully transitioning in a career. Being self-reflective, authentic to one’s identity, and aware are key success factors. There is an opportunity to further evaluate this in future research.

## Conclusion

This literature review analyzed different career strategies and approaches addressing strategic career transition and personal branding. It was determined that career transitions extend beyond the acquisition of new knowledge and the development of new skills and positively impact the formation of new personal brand identities. While the Great Resignation has represented an important point in history in the way people work, it also presented opportunities for transition to different careers. In the words of Barack Obama in his 2008 inauguration speech, “Change will not come if we wait for some other person or if we wait for some other time. We are the ones we have been waiting for. We are the change that we seek.” The change we seek may come through our transition in our careers.

## Author contributions

Both authors listed have made a substantial, direct, and intellectual contribution to the work and approved it for publication.

## Conflict of interest

The authors declare that the research was conducted in the absence of any commercial or financial relationships that could be construed as a potential conflict of interest.

## Publisher’s note

All claims expressed in this article are solely those of the authors and do not necessarily represent those of their affiliated organizations, or those of the publisher, the editors and the reviewers. Any product that may be evaluated in this article, or claim that may be made by its manufacturer, is not guaranteed or endorsed by the publisher.
